# 

**Published:** 1880-12

**Authors:** 


### HYMENEAL.

"Domestic happiness, thou only bliss
Of Paradise that has survived the fall."

Dr. Jud. B. Wood and Miss Bettie J. Davis, of Richmond, Va., only daughter of Mr. Charles T. Davis, member of the city council of Richmond, were married on the 24th of November, ult., at the Carrollton Hotel, in this city. J. W. M. Williams, D. D., of the First Baptist Church, was the officiating minister. The bridesmaids were Misses Lillie and Dora Yearbey, and the groomsmen, Dr. B. M. Wilkerson and Mr. Geo. Savage, Secretary to Mayor Latrobe, all of Baltimore.

There were present the father and mother of the bride, the wife and daughters of the officiating clergyman, E. Calvin Williams, Charles L. Stiel, Prof. Atkinson, Prof. H. L. Byrd, Profs. Gorgas, Harris and Hodgkin. Dr. Wood, the groom, graduated in both medicine and Dentistry in this city, and was for some time a member of the Faculty of his *Alma Mater*, the old Baltimore College of Dental Surgery. He is now practicing his profession in Richmond, his native city, where he has achieved both distinction and success. The happy couple left soon after the ceremony on the southern train for a brief trip to New Orleans and other parts of the sunny South.

### NATALITIAL.

"Of all the joys that brighten suffering earth,
What joy is welcomed like a new-born child?"

Born, in La Fayette, Indiana, on the 7th of November, 1880, a fine boy, to Dr. and Mrs. J. E. Davis, of that city. "The first born of the family."

Born to Dr. and Mrs. Charlie Wilkerson, of Perry Co., Ala., March 5th, 1880, Arthur Foster Wilkerson, their second son.

### SPECIAL NOTICE.

*Dear Sir:*

*We hope that this copy of* **The Practitioner** *will induce you to enroll your name on our subscription list for* 1880, *if you have not already done so. If you do not desire to become a subscriber will you* **please** *do us the* ***kindness*** *to post it back?*

*The January number having been so hastened, it must not be regarded as measuring up to the full standard.*

**The Practitioner** *is handsomely printed on fine book paper, and no labor or expense is spared to make it supply the wants of the earnest practitioner. Each number will contain fifty large octavo pages exclusive of advertisements, making an annual* 600-*page volume of reading matter for* $2.00.

*Hoping that you will fill up the blank on the other side of this sheet, and remit by Postoffice Money Order, Draft or Check at your earliest convenience, we are*

*Very respectfully,*

HARVEY L. BYRD,

BASIL M. WILKERSON.

PROSPECTUS OF THE

### Independent Practitioner:

### A MONTHLY JOURNAL,

*DEVOTED TO MEDICAL, SURGICAL, OBSTETRICAL, DENTAL AND HYGIENICAL SCIENCE.*

The fixed purpose of the projectors of this journal to adhere with unvarying fidelity to independence in all matters appertaining to its conduct and management, renders it unnecessary to issue a lengthy prospectus of its objects and aims. With a few brief statements, therefore, it will be launched forth upon the sea of journalism to take its chances for public favor among the many craft now sailing more or less propitiously thereon. While it respectfully invites subscriptions from every quarter, it naturally looks for primary assistance from old friends and acquaintances. It will know no sectionalism, and, therefore, confidently expects to merit the friendship and good will of those who *really* desire to sustain and keep from degredation to the level of the merest trade, the grandest and noblest calling known to humanity. From all such, subscriptions, contributions on subjects of interest to the profession, and advertisements are respectfully solicited. The experience of the senior editor, in the past, will be brought into activity in catering to the taste of its readers, and nothing will ever mar its pages not consistent with the standard that has been erected for its conduct and management. Being entirely unconnected with colleges and cliques, it will be bold, fearless and honest in speaking and upholding the right in *all* matters coming within its purwiew, and will scrupulously render even-handed justice to every one. All subjects of interest to Physicians, Obstetricians, Surgeons and Dentists will claim the attention of its editors and find space in its pages. To this end translations from the French and German periodicals will be introduced from time to time, and the English and American journals made tributary to its columns. All necessary arrangements have been made to *secure permanency* to the enterprise, and to keep it the independent echo and exponent of all that is new and good in the field of its labors. Exchanges of Medical, Dental and other scientific journals are respectfully requested from all parts of the world. Exchanges and books, except Dental, for notice or review, should be addressed to the senior editor, and Dental exchanges, subscriptions, advertisements, and *all* matters relating to the business management of the journal, should be sent invariably to the junior editor and proprietor.

HARVEY L. BYRD, A. M., M. D.,
*No. 147 Edmondson Avenue.*
BASIL M. WILKERSON, D. D. S., M. D.,
*No. 68 N. Charles Street.*

### SPECIAL NOTICE.

From among a large number of letters commendatory of THE INDEPENDENT PRACTITIONER, we take pleasure in making excerpts from a few from different sections of our country, indicative of the estimate of our labors thus far.

PROF. ISAAC E. TAYLOR, M. D., President of Bellevue Medical College, New York, says "I have been much pleased with the matter introduced, as well as the dress of the Journal, and I wish you all success in the effort to advance the interests of the profession. I noticed a favorable criticism in the *Medical Record* of last week." A few weeks later he gave us a valuable paper which is published in our February issue.

PROF. ROBERTS BARTHOLOW, M. D., of the Jefferson Medical College, Philadelphia, says: "I trust the Journal is succeeding and that you find your editorial work congenial. I shall be pleased to send you a paper when I get through the work which I am compelled to do."

PROF. J. M. DA COSTA, M. D., of the same college and city, writes: "I wish it (the Journal) a most marked success."

PROF. THOS. H. CHANDLER, Dean of the Harvard University, Dental Department, writes: "I am well pleased with your first number. If you keep up the standard, your magazine will fill a void in our professional literature."

PROF. F. PEYRE PORCHER, M. D., Charleston, S. C., writes: "I have read your Journal with pleasure."

PROF. MIDDLETON MICHEL, M. D., of the same city and college, says: "I am much pleased with the appearance and contents of your Journal and wish it success."

PROF. JAS. E. GARRETSON, M. D., D. D. S., Dean of the Philadelphia Dental College, writes: "Let me wish you success in an enterprise which looks as if calculated to be of great use to the dentist. I have read each number of the Journal with much satisfaction and have to apologize for my tardiness in acknowledging your kindness in sending it. I welcome the 'PRACTITIONER' particularly from the standpoint of its *medico*-dental character. The more dentists learn of general medicine and surgery the better are they found qualified for work as specialists."

PROF. J. C. LE HARDY, M. D., of Savannah, Ga., writes: "I am very much pleased with the 'INDEPENDENT PRACTITIONER.'"

PROF. V. H. TALIAFERRO, M. D., of Atlanta, Ga., says: "I will cheerfully contribute my mite to aid in your effort to continue the Journal a grand success."

PROF. W. H. DOUGHTY, M. D., Augusta, Ga., "I wish your enterprise abundant success."

COL. J. J. WOODWARD, Surgeon U. S. Army, "I wish for your enterprise a useful and honorable career and all success."

DR. B. P. ANDERSON, President of the Medical Society of the State of Colorado: "Your Journal is a perfect success. It will give me a great deal of pleasure to assist the 'INDEPENDENT PRACTITIONER' in any way I can."

PROF. WM. A. HAMMOND, M. D., N. Y., and formerly Surgeon-General of the U. S. Army, says: "I am pleased with the 'INDEPENDENT PRACTITIONER,' and wish you success."

PROF. ISAAC J. WETHERBEE, D. D. S., President of the Boston Dental College, writes: "A careful examination of the contents of the 'INDEPENDENT PRACTITIONER' leads me to believe that it will be a very useful publication to the dental profession, and hope that you may meet with large success."

DR. W. R. MUNROE, of Baltimore, "I am happy to receive the 'INDEPENDENT PRACTITIONER,' so neatly and beautifully published, and so ably edited by yourself and colleague. Your prospectus is striking and convincing as to the propriety of your independent position, and I wish you great success in the enterprise you have so nobly inaugurated."

Others among the most intelligent members of the profession in this city have verbally expressed similarly encouraging words.

DR. B. W. HARDEE, of Savannah, Ga., "Merit must and will meet with success."

The foregoing extracts have been taken almost at random from a large number of letters, now before us, and pages could be filled with similar expressions from them.

Space will allow of the mention of a few more names only, of those who have sent us happy greetings or promised articles, viz.: PROF. WM. PEPPER, M. D., PROF. S. WEIR MITCHELL, M. D., DR RICHARD J. DUNGLINSON, PROF. CHAS. J. ESSIG, D. D. S, M. D., Philadelphia; PROF. T. GAILLARD THOMAS, M. D., DR. GEO. M. BEARD, and others, of New York, etc.

We hazard nothing in saying that no medical journal started in this country has been more generally commended, verbally and otherwise, by readers entitled to respect and confidence.

### SPECIAL NOTICE.

From among a large number of letters commendatory of THE INDEPENDENT PRACTITIONER, we take pleasure in making excerpts from a few from different sections of our country, indicative of the estimate of our labors thus far.

PROF. ISAAC E. TAYLOR, M. D., President of Bellevue Medical College, New York, says "I have been much pleased with the matter introduced, as well as the dress of the Journal, and I wish you all success in the effort to advance the interests of the profession. I noticed a favorable criticism in the *Medical Record* of last week." A few weeks later he gave us a valuable paper which is published in our February issue.

PROF. ROBERTS BARTHOLOW, M. D., of the Jefferson Medical College, Philadelphia, says : "I trust the Journal is succeeding and that you find your editorial work congenial. I shall be pleased to send you a paper when I get through the work which I am compelled to do."

PROF. J. M. DA COSTA, M. D., of the same college and city, writes : "I wish it (the Journal) a most marked success."

PROF. THOS. H. CHANDLER, Dean of the Harvard University, Dental Department, writes : "I am well pleased with your first number. If you keep up the standard, your magazine will fill a void in our professional literature."

PROF. F. PEYRE PORCHER, M. D., Charleston, S. C., writes : "I have read your Journal with pleasure."

PROF. MIDDLETON MICHEL, M. D., of the same city and college, says : "I am much pleased with the appearance and contents of your Journal and wish it success."

PROF. JAS. E. GARRETSON, M. D., D. D. S., Dean of the Philadelphia Dental College, write "Let me wish you success in an enterprise which looks as if calculated to be of great use to the dentist. I have read each number of the Journal with much satisfaction and have to apologize for my tardiness in acknowledging your kindness in sending it. I welcome the 'PRACTITIONER' particularly from the standpoint of its *medico*-dental character. The more dentists learn of general medicine and surgery the better are they found qualified for work as specialists."

PROF. J. C. LE HARDY, M. D., of Savannah, Ga., writes : "I am very much pleased with the 'INDEPENDENT PRACTITIONER.'"

PROF. V. H. TALIAFERRO, M. D., of Atlanta, Ga., says : "I will cheerfully contribute my mite to aid in your effort to continue the Journal a grand success."

PROF. W. H. DOUGHTY, M. D., Augusta, Ga., "I wish your enterprise abundant success."

COL. J. J. WOODWARD, Surgeon U. S. Army, "I wish for your enterprise a useful and honorable career and all success."

DR. B. P. ANDERSON, President of the Medical Society of the State of Colorado: "Your Journal is a perfect success. It will give me a great deal of pleasure to assist the 'INDEPENDENT PRACTITIONER' in any way I can."

PROF. WM. A. HAMMOND, M. D., N. Y., and formerly Surgeon-General of the U. S. Army, says : "I am pleased with the 'INDEPENDENT PRACTITIONER,' and wish you success."

PROF. ISAAC J. WETHERBEE, D. D. S., President of the Boston Dental College, writes : "A careful examination of the contents of the 'INDEPENDENT PRACTITIONER' leads me to believe that it will be a very useful publication to the dental profession, and hope that you may meet with large success."

DR. W. R. MUNROE, of Baltimore, "I am happy to receive the 'INDEPENDENT PRACTITIONER,' so neatly and beautifully published, and so ably edited by yourself and colleague. Your prospectus is striking and convincing as to the propriety of your independent position, and I wish you great success in the enterprise you have so nobly inaugurated."

Others among the most intelligent members of the profession in this city have verbally expressed similarly encouraging words.

DR. B. W. HARDEE, of Savannah, Ga., "Merit must and will meet with success."

The foregoing extracts have been taken almost at random from a large number of letters, now before us, and pages could be filled with similar expressions from them.

Space will allow of the mention of a few more names only, of those who have sent us happy greetings or promised articles, viz.: PROF. WM. PEPPER, M. D., PROF. S. WEIR MITCHELL, M. D., DR RICHARD J. DUNGLINSON, PROF. CHAS. J. ESSIG, D. D. S , M. D., Philadelphia ; PROF. T. GAILLARD THOMAS, M. D., DR. GEO. M. BEARD, and others, of New York, etc.

We hazard nothing in saying that no medical journal started in this country has been more generally commended, verbally and otherwise, by readers entitled to respect and confidence.

WILL *YOU* PLEASE SEND IN *YOUR* SUBSCRIPTION MONEY NOW?

### U. S. POSTAL LAW.

:o:

Postmasters are responsible to publishers for the payment of all papers, journals, magazines, etc., not delivered to the persons to whom they are addressed, unless legally accounted for.

Any person who takes a paper from the post-office, whether directed to his name or another, or whether he has subscribed or not, is responsible for the pay.

### SPECIAL INDUCEMENTS

—TO—

### Cash Subscribers.

:o:

We have made arrangements with the editors of the leading scientific, literary and news publications of this country to make a discount of 20 per cent. (and in a few cases more) to cash subscribers to THE INDEPENDENT PRACTITIONER.—Make up your list of magazines and newspapers, *deduct 20 per cent.* (when the discount is more, we will refund it) *from their published rates to single subscribers*, add $2.00 for THE INDEPENDENT PRACTITIONER, Vol. II., 1881, and forward the total amount to us.

This column will be sold for advertising space six month during the year at *special rates*.

N. B.—Each month THE INDEPENDENT PRACTITIONER will be received by a large number of persons for the *first* time, all of whom we trust will be induced to become subscribers. They will please remit NOW the amount of subscription, ($2.00) to insure the continuation of the journal.

